# The impact of JAK/STAT inhibitor ruxolitinib on the genesis of lymphoproliferative diseases

**DOI:** 10.3906/sag-1807-152

**Published:** 2019-04-18

**Authors:** Can TÜRK, Müfide OKAY, Seyhan TÜRK, Elif Sena TEMİRCİ, Osama JAVAD, Salih AKSU, Nilgün SAYINALP, İbrahim Celalettin HAZNEDAROĞLU

**Affiliations:** 1 Department of Medical Microbiology, Faculty of Medicine, Lokman Hekim University, Ankara Turkey; 2 Department of Internal Medicine, Faculty of Medicine, Hacettepe University, Ankara Turkey; 3 Department of Biochemistry, Faculty of Pharmacy, Hacettepe University, Ankara Turkey; 4 Department of Molecular Biology and Genetics, Faculty of Science, Bilkent University, Ankara Turkey

**Keywords:** Lymphoma, ruxolitinib, JAK/STAT signaling pathway

## Abstract

**Background/aim:**

Ruxolitinib, a JAK/STAT signaling pathway inhibitor targeted drug, has been approved for the controlling of disease symptoms and splenomegaly in patients with myeloproliferative neoplastic diseases. Recently, it has been proposed that ruxolitinib-induced JAK/STAT pathway inhibition in myelofibrosis is associated with an elevated frequency of aggressive B-cell lymphomas. However, the biological basis and significance of this pharmacobiological adverse event is unknown. The aim of this bioinformatics study is to detect any possible confounding effects of ruxolitinib on the genesis of lymphoproliferative disorders.

**Materials and methods:**

The gene expression data were retrieved from the E-MTAB-783 Cancer Genome Project database. Gene expression data for all available genes in 26 cell lines belonging to various types of lymphomas were chosen for use in this in silico analysis.

**Results:**

We identified genes that were significant in developing resistance to ruxolitinib in lymphoma cell lines.

**Conclusion:**

Based on the results of our present study, ruxolitinib may potentially lead to the pathological expression of the transcription factors important in lymphoma genesis, neoplastic commitment on the progenitor lymphoid cells, inhibition of repressor transcriptions protective for lymphoma development, inhibition of apoptosis, promotion of neoplastic proliferation, transcriptional activation, and proliferation of malignant neoplastic B cells.****

## 1. Introduction 

Ruxolitinib, a novel oral JAK 1 and 2 inhibitor, was newly certified as a revolutionary treatment for patients suffering from intermediate/high risk myelofibrosis (1). It was demonstrated that the exposure of cutaneous T-cell lymphoma cell lines triggered a dose-dependent inhibition of cell proliferation by a mechanism that impacted the control of DNA synthesis, with a significant decline in the basal levels of phospho-STAT3 (2). 

Furthermore, the ruxolitinib-mediated decrease in circulating proinflammatory cytokine levels is connected to its inhibitory effects on JAK1-mediated signaling; this may make ruxolitinib a tempting therapeutic agent for MYD88 mutation-positive lymphoma patients, as this mutation leads to cell-autonomous activation of JAK 1 and JAK 2 (3). JAK/STAT signaling has been detected to be vital in the mammary gland, lymphocytes, adipocytes, neuronal cells, cardiomyocytes, hepatocytes, eye cells, and stem cells. Deregulation in the JAK/STAT pathway is suggested as a cause of numerous diseases including the emergence and the advancement of cancer cells (4). 

The JAK/STAT pathway controls embryonic development and is also a part of the regulation of many processes such as stem cell maintenance, hematopoiesis, and the inflammatory response (5). STATs have critical as well as nonredundant functions in lymphocyte development. They impact cell fate decisions like differentiating naive T cells, regulate the intensity and period of inflammatory responses, and contributes to pathogenic mechanisms in chronic inflammatory diseases. STAT3 governs the differentiation of naive T cells into the regulatory and inflammatory T-cell lineages. It also controls cell growth, apoptosis, and the transcription of inflammatory genes, and it contributes to the development of chronic inflammatory diseases, as well as malignant and neurodegenerative diseases (5). STATs are also known to control counteracting cellular events by contributing to the induction of apoptosis, as well as in differentiation and stem cell maintenance. STAT3 signaling throughout mammary gland involution induces epithelial cell death. In opposition to this, persistent and enhanced STAT3 activation is an initiator of tumorigenesis (5). Moreover, JAK/STAT signaling leads to immunosuppression and controls inflammatory responses, obesity, stem cell maintenance, and the premetastatic niche development. These effectors and the direct and mediated mechanisms of JAK/STAT signaling in and on tumors cells improved our outlooks for JAK/STAT-based cancer therapeutics (5). 

The aim of this bioinformatics study is to detect any possible confounding effects of ruxolitinib on the genesis of lymphoproliferative disorders. Recently, it has been proposed that ruxolitinib-induced JAK/STAT pathway inhibition in myelofibrosis is associated with an elevated frequency of aggressive B-cell lymphomas (6). Elucidation of this unique drug acting on multiple biological downstream paths is important since it is already accepted as a standard medication for the real-life management of patients with chronic myeloproliferative disorders, namely myelofibrosis and polycythemia vera (PV) (7,8). The management of such patients is based on randomized clinical trials such as the COMFORT-I, COMFORT-II, and RESPONSE trials and real-life administration data (9–11). However, chronic myeloproliferative disorders are relatively benign diseases in comparison to the acute myeloproliferation. Therefore, there is a general safety concern for the long-term administration of ruxolitinib, particularly for the emergence of secondary malignancies such as lymphomas (9,10). Hence, the findings of this study can underline the complication of lymphoma development with the probable relative long-term usage of ruxolitinib as a JAK/STAT signal transduction inhibitor (6). 

## 2. Materials and methods 

### 2.1. Microarray gene expression and drug cytotoxicity data 

The gene expression data used in this study were retrieved from the E-MTAB-783 Cancer Genome Project (CGP) database and RMA-normalized (12). The database contained data for 13,513 genes, amounting to 22,279 probe sets. The expression of these genes in 773 cell lines, belonging to a variety of cancer types, was available. Out of these, gene expression data for all available genes in 26 cell lines belonging to various types of lymphomas (B-cell lymphoma, Burkitt lymphoma, and Hodgkin lymphoma) were chosen for use in in silico analysis. The drug cytotoxicity data for ruxolitinib were obtained from the E-MTAB-783 CGP database for drug sensitivity (12). 

### 2.2. Identification of the genes whose expressions correlated with ruxolitinib sensitivity 

The previously retrieved and RMA-normalized gene expression data for 13,513 genes in 26 lymphoma cell lines (B-cell lymphoma, Burkitt lymphoma, and Hodgkin lymphoma) were correlated with drug cytotoxicity data for ruxolitinib. Using Pearson correlation analysis, genes that could be associated with ruxolitinib sensitivity were identified.

 From this analysis, genes with the highest correlation were then used for further analysis. A list of genes containing all the genes that we were especially interested in investigating was compiled. The selection criteria of genes included clinicopathological correlation of lymphoma genesis and ruxolitinib administration. During the selection of genes, a clinician and molecular biologist worked together in order to determine clinically relevant and biologically important genes to underline the research endpoints. These genes are henceforth referred to as genes of interest in this study and are compiled in Table 1. This list had 200 genes; 155 belonged to the JAK/STAT pathway, 31 were cancer stem cell (CSC) markers, and 14 were immune response markers. 

**Table 1:** List of genes of interest used as the basis of this study.

Genes of interest		
Total = 200		
JAK/STAT Genes*	Cancer stem cell (CSC) markers**	Immune response markers***
Total = 155	Total = 31	Total = 14
GH1, TIMP-1, CSH1, PIK3CB, PIAS3, MPL, APP, IFNAR1, IL6R, AKT1, AKT2, AKT3, AP1, BCL2L1, CBL, CBLB, CBLC, CCND1, CCND2, CEBPB, CISH, CLCF1, CNTF, CNTFR, CREBBP, CRLF2, CSF2, CSF2RA, CSF2RB, CSF3, CSF3R, CTF1, EGF, EGFR, EP300, EPO, EPOR, ERK, GH2, GHR, GRB2, IFNA1, IFNA14, IFNA17, IFNA2, IFNA21, IFNA4, IFNA5, IFNA6, IFNA8, IFNAR2, IFNB1, IFNG, IFNGR1, IFNGR2, IFNW1, IL10, IL10RA, IL10RB, IL11RA, IL12A, IL12B, IL12RB1, IL12RB2, IL13,	CD22, CD27, CD19, CD20, CD40, CD45, ALDH1A1, CCND1, CD10, CD133, CD138, CD15, CD23, CD25, CD30, CD34, CD4, CD43, CD44, CD5, CD7, CD71, CD77, CD80,	CD278, CD279, CD3D, CD3E,CD3G, CD4, CD47, CD68, CD8, CTLA4, FoxP3, IDO-1,LAG3, MUM1
IL13RA1, IL13RA2, IL15, IL15RA, IL19, IL2, IL20RA, IL21, IL21R, IL22, IL22RA1, IL23A, IL24, IL26, IL2RA, IL2RB, IL2RG, IL3, IL3RA, IL4, IL4R, IL5, IL5RA, IL6, IL6ST, IL7, IL7R, IL9, IL9R, IRF9, JAK1, JAK2, JAK3, LEP, LEPR, LIF, LIFR, Mcl-1, MEK2, MTOR, MYC, NFKB1, NFKB2, OSMR, P120, PIAS1, PIAS2, PIAS4, PIK3CA, PIK3CD, PIK3CG, PIK3R1, PIK3R2, PIK3R3, PIK3R5, PIM1, PRL, PRLR, PTPN1, PTPN11, PTPN2, PTPN6, RAF1, SHC1, SOCS1, SOCS2, SOCS3, SOCS5, SOCS7, SOS1, SOS2, SPRED2, SPRY1, SPRY2, SPRY4, SRC, STAM, STAM2,STAT1, STAT2, STAT3, STAT5A, STAT5B, STAT6, SUMO1, TF, TPO, TYK2	CD8A, LMP1, MMP2, MMP7, MMP9, TGFB1	

*Genes that are a part of the JAK/STAT mediated signaling pathway. **Genes that are known to be cancer stem cell markers within lymphoma. ***Genes belonging to the immune response pathway that are identified as markers for lymphoma.

The highly correlated genes from the Pearson correlation analysis were then searched to see if any of them were genes of interest. Following this, the top 10 directly correlated genes and top 10 inversely correlated genes that were highly correlated were selected. Lymphoma cell lines were hierarchically clustered based on these genes, using Gene Cluster 3.0 with Euclidean distance as a similarity metric parameter for genes and arrays, and complete linkage. 

### 2.3. Determination of differentially expressed genes within resistant and sensitive groups 

We had gene expression data for 26 lymphoma cell lines. We decided to divide these cell lines into two groups based on IC50 values for ruxolitinib to see if we could distinguish between cell lines that were sensitive to ruxolitinib and cell lines that were resistant to ruxolitinib. 

 A bar chart and a heat-map were generated in Excel from the IC50 values to visualize the increase in IC50 values across the 26 cell lines. A two-tailed, unpaired t-test was performed between cell lines with the lowest IC50 values, the sensitive group (HDLM2, TUR, BC3), and cell lines with the highest IC50 values, the resistant group (RAJI, DG75, RPMI6666, DB). P < 0.05 was determined to be an appropriate standard for selection of the two groups. 

The 200 genes of interest, containing 155 JAK/STAT pathway genes, 31 CSC markers, and 14 immune response markers, were now analyzed within the sensitive group (HDLM2, TUR, BC3) and resistant group (RAJI, DG75, RPMI6666, DB). A two-tailed, unpaired t-test was used to verify which genes were differentially expressed in the two groups. A cut-off of 0.05 was used for the P-values. 

Separate hierarchical clusters were first created for all 26 lymphoma cell lines and then for only the 7 cell lines in the two groups, based on the significant differentially expressed genes. The clustering was done using Gene Cluster 3.0 with Euclidean distance as a similarity metric parameter for genes and arrays, and complete linkage. 

### 2.4. Gene-set enrichment analysis 

The gene expression data for all 13,513 genes found in the E-MTAB-783 CGP database, and for the 7 cell lines in the sensitive group (HDLM2, TUR, BC3) and resistant group (RAJI, DG75, RPMI6666, DB), were put into GSEA 3.0 to determine which genes were significantly different within the two groups. Furthermore, we wanted to highlight the pathways that these genes were a part of in both these groups (12). 

### 2.5. Network analysis 

Out of the 200 genes of interest, the 25 genes that were differentially expressed were used as input for the GeneMANIA app (13) in Cytoscape (14) to generate a network based on coexpression and genetic interactions. That was done in order to find if the two sets have functionally similar genes related to each other and to determine the associated functions for different groups of genes in the network. The network served as a starting point for the pathway analysis approach in Reactome (15) to filter out regulatory pathways that are relevant for both these gene sets. 

## 3. Results 

We identified genes that were significant in developing resistance to ruxolitinib in lymphoma cell lines. We used a statistical approach that allowed us to correlate the IC50 data for ruxolitinib and gene expression for desired genes and create a linear relationship between the two. 

### 3.1. Genes correlated with ruxolitinib sensitivity 

**Figure 1:**
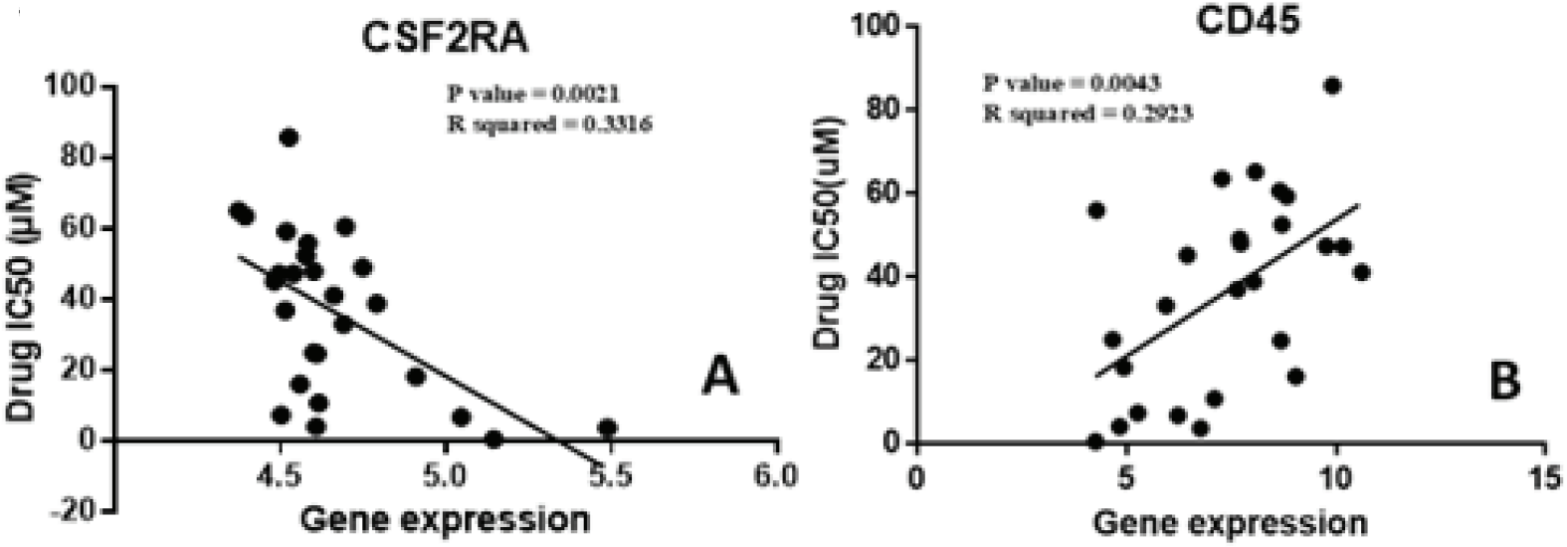
Identification of highly correlated genes. Among the genes that were analyzed with ruxolitinib drug cytotoxicity data, CSC biomarkers and JAK/STAT pathway genes were among the highly correlated. CD45, A, shows the highest direct correlation among the CSC biomarkers with an r value of 0.54. CSF2RA, B, shows the highest inverse correlation amongst the JAK/STAT pathway genes with an r value of –0.58.

**Figure 2:**
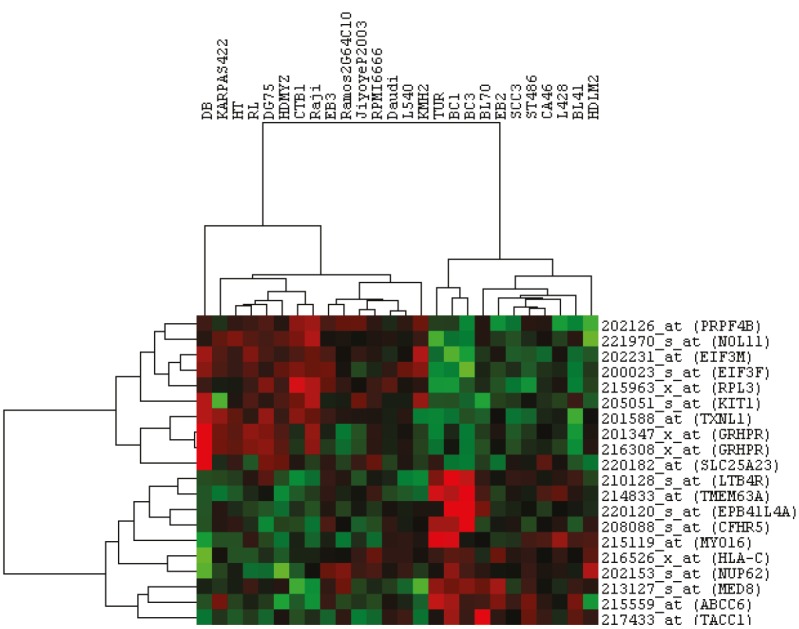
Highly correlated genes show the presence of distinct groups within lymphoma. The top ten directly correlated genes along with the top ten inversely correlated genes were hierarchically clustered with all available lymphoma cell lines. All gene expressions used were standardized.

The gene expression data for all 13,513 genes in the E-MTAB-783 CGP database for lymphoma cell lines were analyzed (12). By doing Pearson correlation analysis with these genes, we were able to find the most highly correlated genes with ruxolitinib sensitivity. A cut-off of 0.4 was used for the R-values so that a large number of genes could be considered. With this cut-off, 800 genes were shown to be highly correlated with a direct relationship while 584 were shown to be highly correlated with an inverse relationship. Further analyses were done using these highly correlated genes. Of these highly correlated genes, we found a number of genes that were of interest to us. We had compiled a list of 200 genes for this study that we wanted to investigate. The list contained 155 JAK/STAT pathway genes, 31 CSC markers, and 14 immune response markers. Our analysis showed that 4 JAK/STAT pathway genes had a direct relationship with ruxolitinib resistance, while 8 JAK/STAT pathway genes showed an inverse relationship. Figure 1A shows one of the genes, *CSF2RA*, which has an inverse relationship with the development of ruxolitinib resistance. On the other hand, we also found a direct linear relationship between the expressions of 5 CSC markers and resistance to the drug. Figure 1B shows the correlation of one of these genes, *CD45*. Unfortunately, no immune response markers were found among the highly correlated genes. Following this, the ten highest directly correlated genes and the ten highest inversely correlated genes were hierarchically clustered. The results shown in Figure 2 show the presence of two distinct clusters within lymphoma cell lines. 

### 3.2. Determination of sensitive and resistant groups within lymphoma cell lines 

The 26 available lymphoma cell lines were divided into two groups, where one group was significantly more resistant to ruxolitinib than the other. Significance was determined by performing a two-tailed, unpaired t-test between the groups and accepting P-values of less than 0.05. 

**Figure 3:**
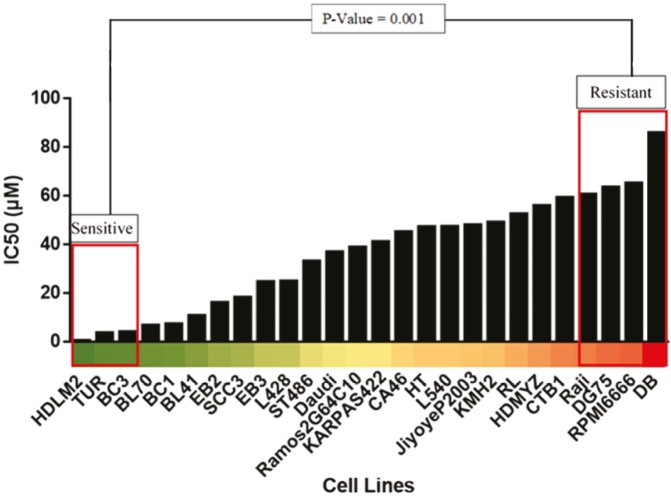
The sensitive and resistant groups show significant changes in ruxolitinib IC50. The cell cytotoxicity data for ruxolitinib available in the E-MTAB-783 CGP database were compared for each lymphoma cell line. The bar chart above shows the vast range of IC50 for lymphoma cell lines, which allowed us to create two distinct groups. The selected groups were significant with a P-value of 0.001 (20).

IC50 data for ruxolitinib were plotted for each cell line available. As a result, a comparative bar chart was created, as shown in Figure 3. This allowed for the clear determination of the level of sensitivity of each cell line to ruxolitinib. A heat-map was created to further highlight the range of sensitivity to ruxolitinib in lymphoma cell lines, also seen in Figure 3. Based on the bar chart and the heat-map, the cell lines were divided into two groups. The sensitive group (HDLM2, TUR, BC3) included the cell lines that corresponded to the lowest ruxolitinib IC50 values, hence being the most sensitive to this drug. The resistant group (RAJI, DG75, RPMI6666, DB) included the cell lines that corresponded to the highest ruxolitinib IC50 values, being the least sensitive and most resistant to action of this drug. The resistant group, compared to the sensitive group, was significantly more resistant to ruxolitinib, with a P-value of 0.001. 

### 3.3. Differentially expressed genes within resistant and sensitive groups 

**Figure 4:**
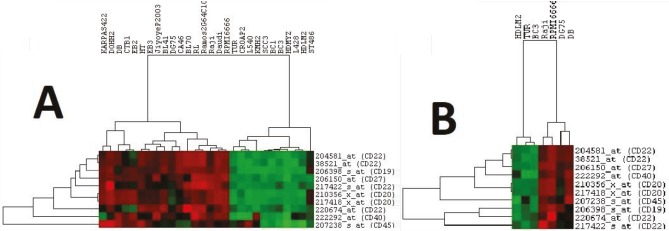
Clustering of lymphoma cell lines with differentially expressed CSC markers. The CSC markers that were differentially expressed were first hierarchically clustered with all available lymphoma cell lines as shown in A. These same genes were then clustered with only the sensitive and resistant group cell lines, as shown in B. In both cases two major clusters can be seen, one with sensitive cells and the other with resistant cells.

**Figure 5:**
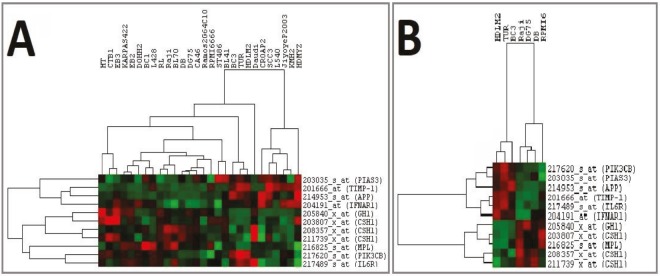
Clustering of lymphoma cell lines with differentially expressed JAK/STAT pathway genes. Significant JAK/STAT pathway genes were clustered with all available lymphoma cell lines, shown in A. These genes were then clustered with only the cell lines from the sensitive and resistant groups, as shown in B.

The E-MTAB-783 CGP database for gene expression in cancer cell lines was used with a focus on the lymphoma cell lines that were part of the resistant and sensitive groups (12). Analysis of the comparative gene expression between these two groups revealed that 904 genes were differentially expressed, with P-values of less than 0.05. The results further showed that out of these 904 genes, 15 belonged to the list of 200 genes of interest that we had compiled for this study. These 15 genes are displayed in Table 2 along with their classifications; 9 of these genes are involved in the JAK/STAT pathway while 6 are CSC markers. These genes and their clinical significance are shown in Table 3. This allowed us to form a relationship between the expression of genes of interest and ruxolitinib sensitivity. To further demonstrate this relationship, differentially expressed genes for the JAK/STAT pathway and CSC markers were hierarchically clustered, as shown in Figure 4 and Figure 5, respectively. These figures can be seen having two distinct clusters. 

**Table 2:** List of genes of interest that are differentially expressed within resistant and sensitive groups.

Significant genes	
JAK/STAT pathway	CSC markers
GH1 (P = 0.0066)	CD22 (P = 0.0170)
TIMP-1 (P = 0.0181)	CD27 (P = 0.0013)
CSH1 (P = 0.0342)	CD19 (P = 0.0035)
PIK3CB (P = 0.0224)	CD20 (P = 0.0145)
PIAS3 (P = 0.0312)	CD40 (P = 0.0228)
MPL (P = 0.0316)	CD45 (P = 0.0275)
APP (P = 0.0339)	
IFNAR1 (P = 0.0339)	
IL6R (P = 0.0376)	

**Table 3:** List of lymphoid cancer stem cell genes that are affected via ruxolitinib usage and their clinical correlations.

Gene*	Physiological function**	Pathobiological alteration**	Possible disordered mechanism**	Related lymphoidneoplasms**
B-Cell related genes*CD19CD20CD23CD25CD22	B-lymphocyte development, growth, differentiation, determination of B-cell functions	Neoplastic aberrations resulting in numerous lymphoid malignant disorders	Pathological expression of the transcription factors, proliferation of malignant neoplastic B cells	T-cell lymphoma (21)B-cell lymphoma (22,23)Hodgkin lymphoma (24,25)Follicular lymphoma (26,27)
T-Cell related genes*CD5CD10LMP-1	T-cell proliferation, inactivation of critical peptide hormones important for lymphoid functions	Pathological alterations of transmembrane glycoproteins and lymphoid cell surface markers	Inhibition of cellular apoptosis	CLLSLLMCL (29)Nasal NK/T-cell lymphoma (30)
Lymphoid / lineage-related genes*CD45CD30CD15CD79CD138CD38CD43CD71	Regulation of signaling, lymphoid cellular processes including cell growth, differentiation, mitosis, oncogenic transformation, and immune activation	Pathological alterations of cellular proliferation, migration, and cell–matrix interactions	Neoplastic proliferation of B-lymphoid cells andplasma cells	DLBCL (31), NK/T-cell lymphoma (32), AITL (33), PTCL (NOS) (34),Hodgkin lymphoma (34),PTCL (35),extranodal NK/T-cell lymphoma (36),DLBCL (37), DLBCL (37), ATLL (20)

*Genes affected by ruxolitinib treatment. **Selected physiological actions, pathobiological alterations, possible mechanisms for the genesis of lymphoid neoplasms. CLL: Chronic lymphocytic leukemia, SLL: small lymphocytic lymphoma, MCL: mantle cell lymphoma, DLBCL: diffuse large B cell lymphoma, AITL: adult T-cell leukemia/lymphoma, PTCL: Peripheral T-cell lymphoma, ATLL: adult T-cell leukemia/lymphoma.

### 3.4. Gene-set enrichment analysis 

Gene-set enrichment analysis was then performed between the sensitive and resistant groups of the lymphoma cell lines. The analysis was performed with all the genes available in the E-MTAB-783 CGP database (12). The results highlighted the genes that showed significant differences between the two groups and provided us with insight about which pathways these significant genes were involved in. Table 4 and Table 5 respectively show the pathways that were found to be statistically noteworthy, with P-values of less than 0.05, within the sensitive and the resistant groups.

**Table 4:** List of pathways significant within the sensitive group.

	GENESET PATHWAY	SIZE	NOM P	FDR q
1	Go_Enzyme_Inhibitor_Activity	19	0	0.013
2	Go_Secretory_Vesicle	32	0	0.064
3	Go_Secretory_Granule	27	0	0.157
4	Go_Vacuolar_Membrane	36	0	0.215
5	Go_Cell_Leading_Edge	26	0.002	0.232
6	Go_Vacuolar_Part	43	0.003	0.095
7	Go_Protein_Complex_Binding	53	0.003	0.189
8	Go_Vacuole	65	0.003	0.279
9	Go_Oxidation_Reduction_Process	45	0.005	0.321
10	Go_Transition_Metal_Ion_Binding	67	0.005	0.268
11	Go_Vesicle_Mediated_Transport	69	0.009	0.257
12	Go_Membrane_Microdomain	20	0.013	0.269
13	Go_Transport_Vesicle	24	0.014	0.261
14	Go_Golgi_Apparatus	78	0.014	0.393
15	Go_Lipid_Localization	18	0.015	0.3
16	Go_Lytic_Vacuole	31	0.016	0.282
17	Go_Hydrolase_Activity_Acting_On_Acid_Anhydrides	43	0.016	0.29
18	Go_Cytoskeletal_Protein_Binding	45	0.016	0.267
19	Go_Cytoskeleton	80	0.017	0.277
20	Go_Sulfur_Compound_Metabolic_Process	20	0.018	0.274
21	Go_Macromolecular_Complex_Binding	67	0.018	0.396
22	Go_Zinc_Ion_Binding	57	0.019	0.335
23	Go_Regulation_Of_Lipid_Metabolic_Process	18	0.022	0.276
24	Go_Locomotion	59	0.022	0.392
25	Go_Presynapse	17	0.024	0.327
26	Go_Golgi_Apparatus_Part	47	0.03	0.272
27	Go_Enzyme_Regulator_Activity	41	0.03	0.387
28	Go_Endocytosis	32	0.037	0.391
29	Go_Positive_Regulation_Of_Cytokine_Production	24	0.037	0.384
30	Go_Golgi_Membrane	36	0.042	0.408

**Table 5:** List of pathways significant within the resistant group.

	GENESET PATHWAY	SIZE	NOM P	FDR q
1	ncRNA Processing	40	0	0
2	Ribosome Biogenesis	37	0	0
3	ncRNA Metabolic Process	44	0	0
4	RNA Catabolic Process	33	0	0
5	Organic Cyclic Compound Catabolic Process	40	0	0.001
6	Ribonucleoprotein Complex Biogenesis	42	0	0.001
7	rRNA Metabolic Process	35	0	0.001
8	Nuclear Transcribed mRNA Catabolic Process Nonsense Mediated Decay	27	0	0.002
9	Structural Constituent Of Ribosome	27	0	0.002
10	Translational Initiation	26	0	0.004
11	Ribonucleoprotein Complex	45	0	0.004
12	Cytosolic Part	25	0	0.005
13	Ribosomal Subunit	25	0	0.005
14	Protein Targeting To Membrane	27	0	0.005
15	Posttranscriptional Regulation Of Gene Expression	27	0	0.005
16	Amide Biosynthetic Process	40	0	0.005
17	Ribosome	27	0.002	0.006
18	Protein Localization To Endoplasmic Reticulum	26	0	0.009
19	Establishment Of Protein Localization To Endoplasmic	26	0	0.011
20	Cytosolic Ribosome	24	0	0.012
21	Regulation Of Cellular Amide Metabolic Process	24	0.003	0.015
22	RNA Processing	63	0	0.016
23	B Cell Activation	15	0	0.02
24	Immune Response Regulating Cell Surface Receptor	19	0	0.021
25	Multi Organism Metabolic Process	26	0.002	0.021
26	RNA Binding	100	0.001	0.024
27	Establishment Of Protein Localization To Membrane	31	0	0.038
28	Poly A RNA Binding	83	0	0.039
29	mRNA Metabolic Process	53	0	0.039
30	Structural Molecule Activity	46	0.003	0.086

###  3.5. Network analysis

**Figure 6:**
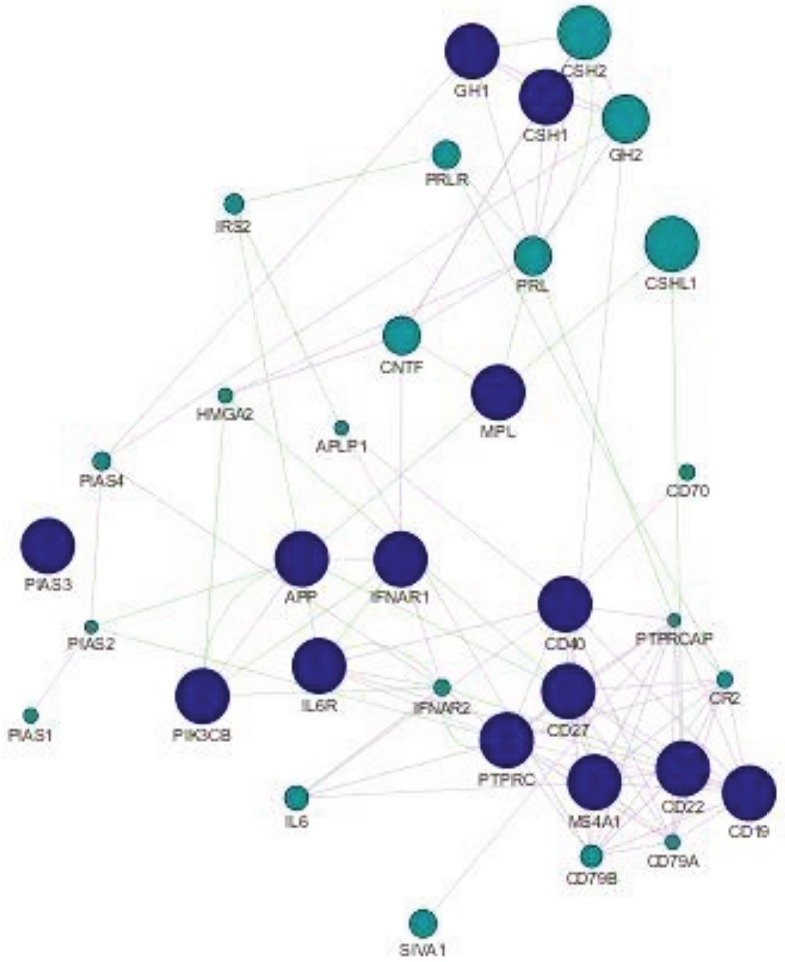
GeneMANIA generated network showing interaction patterns of JAK/STAT pathway and CSC marker gene sets based on coexpression and genetic interactions. Input genes are shown in blue (14 nodes), while the ones identified as related by the program are shown in green. The coexpression-based interactions are shown by purple edges, while the ones from genetic interactions are shown in green.

Our previous analysis had revealed that 15 of 200 genes of interest were differentially expressed in the sensitive and resistant groups. Nine of these belonged to the JAK/STAT pathway while the other 6 were CSC markers. These 15 genes were input into the GeneMANIA app in Cytoscape to generate a network based on coexpression and genetic interactions. Cytoscape tools were used to differentiate the input genes from both sets from the ones that GeneMANIA predicts as likely to share the same function based on their interactions, as seen in Figure 6. From the network, it is distinctly clear that the two sets of genes are connected with one another. Furthermore, the results of GeneMANIA analysis provided a list of associated functions for different groups of genes in the network, as shown in Table 6. The integration of different genes together with their function allows for a complete view of potential regulatory mechanisms occurring in a biological process. Reactome analysis was then used to group genes based on their common pathways, demonstrated in Table 7. 

**Table 6:** The list of functions associated with differently grouped genes in the network.

GO ID	Description	q-value	Occurrencesin Sample	Occurrencesin Genome
GO:0007259	JAK-STAT cascade	3.24E-18	13	91
GO:0046427	positive regulation of JAK-STAT cascade	1.04E-08	7	45
GO:0060397	JAK-STAT cascade involved in growth hormone signaling pathway	1.48E-08	6	24
GO:0071378	cellular response to growth hormone stimulus	4.01E-08	6	31
GO:0060396	growth hormone receptor signaling pathway	4.01E-08	6	31
GO:0046425	regulation of JAK-STAT cascade	4.01E-08	7	61
GO:0060416	response to growth hormone	4.22E-08	6	32
GO:0042113	B cell activation	5.71E-08	8	119
GO:0042100	B cell proliferation	9.11E-07	6	54
GO:0046651	lymphocyte proliferation	2.97E-06	7	123
GO:0032943	mononuclear cell proliferation	3.03E-06	7	125
GO:0070661	leukocyte proliferation	4.29E-06	7	133
GO:0042531	positive regulation of tyrosine phosphorylation of STAT protein	8.43E-06	5	38
GO:0042509	regulation of tyrosine phosphorylation of STAT protein	1.68E-05	5	44
GO:0007260	tyrosine phosphorylation of STAT protein	2.46E-05	5	48
GO:0097285	cell-type specific apoptotic process	4.42E-05	7	194
GO:0018108	peptidyl-tyrosine phosphorylation	6.75E-05	7	210
GO:0042517	positive regulation of tyrosine phosphorylation of Stat3 protein	6.75E-05	4	23
GO:0018212	peptidyl-tyrosine modification	6.75E-05	7	210
GO:0050730	regulation of peptidyl-tyrosine phosphorylation	1.23E-04	6	138
GO:0016925	protein sumoylation	1.40E-04	4	28
GO:0050871	positive regulation of B cell activation	1.42E-04	4	29
GO:0071375	cellular response to peptide hormone stimulus	1.42E-04	7	244
GO:0042516	regulation of tyrosine phosphorylation of Stat3 protein	1.42E-04	4	29
GO:0005126	cytokine receptor binding	1.43E-04	6	148
GO:1901653	cellular response to peptide	1.43E-04	7	247
GO:0042503	tyrosine phosphorylation of Stat3 protein	1.63E-04	4	31
GO:0043434	response to peptide hormone	1.63E-04	7	255
GO:1901652	response to peptide	1.80E-04	7	260

**Table 7:** The resulting pathways from Reactome analysis and the number of genes for each of them ordered by P-value.

Pathway identifier	Pathway name	#Entities found	EntitiespValue
R-HSA-1280215	Cytokine Signaling in Immune system	10	1.55E-07
R-HSA-168256	Immune System	13	1.39E-06
R-HSA-112411	MAPK1 (ERK2) activation	2	1.13E-04
R-HSA-110056	MAPK3 (ERK1) activation	2	1.33E-04
R-HSA-1059683	Interleukin-6 signaling	2	2.27E-04
R-HSA-1170546	Prolactin receptor signaling	2	2.54E-04
R-HSA-112409	RAF-independent MAPK1/3 activation	2	6.10E-04
R-HSA-982772	Growth hormone receptor signaling	2	6.54E-04
R-HSA-6783589	Interleukin-6 family signaling	2	7.00E-04
R-HSA-8862803	Deregulated CDK5 triggers multiple neurodegenerativepathways in Alzheimer’s disease models	2	7.46E-04
R-HSA-8863678	Neurodegenerative Diseases	2	7.46E-04
R-HSA-449147	Signaling by Interleukins	5	10.80E-03
R-HSA-76002	Platelet activation, signaling and aggregation	3	57.84E-03
R-HSA-2219530	Constitutive Signaling by Aberrant PI3K in Cancer	2	66.73E-03
R-HSA-198933	Immunoregulatory interactions between a Lymphoid and a non-Lymphoid cell	3	74.69E-03
R-HSA-1280218	Adaptive Immune System	5	75.00E-03
R-HSA-5668541	TNFR2 non-canonical NF-kB pathway	2	79.43E-03

## 4. Discussion 

The JAK-STAT pathway may play an important role in the pathogenesis of lymphoma (2,16). In humans, the activator of the JAK-STAT family comprises four JAKs (JAK 1, JAK 2, JAK 3, and tyrosine kinase 2 (TYK 2)) and seven STATs (STAT 1, STAT 2, STAT 3, STAT 4, STAT 5a, STAT 5b, STAT 6) proteins (5). JAK-STAT proteins play a key role in regulating lymphoid homeostasis and immunity. This includes the maintenance of the balance between the development of T helper 1 (Th1) and T helper 2 (Th2), T-cell response, regulatory T (Treg) cells, and the function of memory CD8+ cells in myeloid and lymphoid development (5). JAK-STAT signaling is perversely activated in lymphoma by multiple mechanisms, together with inappropriate autocrine and paracrine cytokine stimulation. Genetic and epigenetic changes of negative regulators of JAK/STAT signaling, such as loss of function, SOCS1 mutations, and deletions of the protein tyrosine phosphatase 2 (PTPN2), may also cause deregulated JAK/STAT initiation in Hodgkin lymphoma, primary mediastinal large B-cell lymphoma, diffuse large B-cell lymphoma, follicular lymphoma, and Peripheral T-cell lymphoma (13). As mentioned, one of the foremost pathways taking part in the signal transductions of a wide array of these cytokines is the JAK/STAT cascade. The JAK/STAT pathway is crucial for many significant biological processes, including broad immune and hematopoietic cell functions (1). 

Acquired mutations may influence the JAK/STAT pathway by activating members of the JAK and STAT families directly, inactivating proteins whose typical function is to deactivate the JAKs, or establishing autocrine signaling loops that drive JAK-mediated multiplication. Some of the mutations that are found to be linked to lymphoma development directly target genes that encode elements of the JAK/STAT pathway. JAK 1 and STAT 3 mutations show this relationship between lymphoma development and the JAK/STAT pathway (2). However, even in the presence of STAT or JAK mutations, the whole functional cytokine receptors, JAK and STAT, were required to maintain activation and malignant cell proliferation (17). STAT 3 and STAT 5b mutations are present in aggressive lymphomas emerging from natural killer cells as well as the mutated STATs that are related to increased tyrosine phosphorylation. They supply a growth benefit to natural killer cells, which can be partially inhibited by a JAK 1 and JAK 2 inhibitor (4). 

Preliminary studies considering the efficacy of JAK inhibitors illustrate that therapeutic agents targeting the JAK/STAT signaling pathway can be used to treat patients with lymphoma (3). On the other hand, ruxolitinib-induced JAK/STAT pathway inhibition in myelofibrosis is associated with an elevated frequency of aggressive B-cell lymphomas (6). Based on the results of our present in silico study, there must be some concern for the development of lymphoproliferative neoplastic disease in a given patient under ruxolitinib since the drug affects numerous genes that have a clear impact on the pathobiology of lymphomas. Table 3 summarizes the list of genes present in lymphoid cancer stem cells that were affected by ruxolitinib usage and their clinical correlations. Thus, ruxolitinib may potentially lead to the pathological expression of the transcription factors important in lymphomagenesis, neoplastic commitment on the progenitor lymphoid cells, inhibition of repressor transcriptions protective for lymphoma development, inhibition of apoptosis, promotion of neoplastic proliferation, transcriptional activation, and proliferation of malignant neoplastic B cells (Table 3). Hence, we fully agree with Porpaczy et al. that the detection of a preexisting B-cell clone may identify individuals at risk for lymphoma development and any ruxolitinib candidate should undergo a bone marrow biopsy procedure for the evaluation of clonal lymphoid baseline proliferation (6). This suggestion is particularly helpful for early stage myelofibrosis or PV since those patients potentially have decades for survival (7,18,19). Ruxolitinib is administered for PV just for the control of symptoms, but if lymphoma due to ruxolitinib complicates the clinical picture, the survival could be decreased from about two decades to several months in a given PV patient. 

Ruxolitinib is a JAK/STAT signaling pathway inhibitor targeted drug with predictable pharmacobiological actions. The main function of the JAK/STAT signaling pathway is cellular proliferation in health and disease. The drug has been approved for the treatment of patients with high- or intermediate-risk myelofibrosis (MF) with symptomatic splenomegaly. The development of lymphoma due to ruxolitinib might be acceptable for a high-risk advanced stage of MF. However, based on the results of our current study, the administration of ruxolitinib may not be rational for symptom control only in early-stage MF, low-risk MF, and PV patients for the potential pathobiological risk of lymphoma development. 

The clinical relevance of the novel results of our study adds support to the concerns of Porpaczy et al. (6). If pharmacobiological aspects of ruxolitinib are related to the danger of the development of lymphoma in patients with chronic myeloproliferative disorders, then decades of usage of the drug could be harmful at least for the patient subpopulation with already present preneoplastic bone marrow lymphoid follicles. Clinicians dealing with the management of chronic myeloproliferative disorders should be aware of this fact and should clinicopathologically check lymphoid neoplastic disorders before and during the administration of ruxolitinib. Of course, myeloid and lymphoid neoplasia are different diseases, but long-term administration of JAK/STAT inhibitors may be located at a crossroads to complicate lymphoma in a patient with chronic myeloproliferative disease.

The development of any drug from bench side to the clinic is very difficult, painful, and expensive. Sometimes unexpected pharmacobiological adverse effects could complicate the treatment strategy with novel drugs. Therefore, a well-developed scientific strategy is absolutely necessary for the design of clinical studies at all critical points. Ruxolitinib is the first clinically useful targeted therapy in Ph*-negative MPNs. In the interest of a clinically advanced approach to the study of this drug, the progression of lymphoma development associated with ruxolitinib in addition to the disease risk profile and scoring of MPNs should be included.
